# Establishing the Relationship between Cutting Speed and Output Parameters in Belt Grinding on Steels, Aluminum and Nickel Alloys: Development of Recommendations

**DOI:** 10.3390/ma14081974

**Published:** 2021-04-15

**Authors:** Nelli Vladimirovna Syreyshchikova, Danil Yurievich Pimenov, Munish Kumar Gupta, Krzysztof Nadolny, Khaled Giasin, Shubham Sharma

**Affiliations:** 1 Department of Automated Mechanical Engineering, South Ural State University, Lenin Prosp. 76, 454080 Chelyabinsk, Russia; snv.ktn@mail.ru (N.V.S.); munishguptanit@gmail.com (M.K.G.); 2 Key Laboratory of High Efficiency and Clean Mechanical Manufacture, Ministry of Education, School of Mechanical Engineering, Shandong University, Jinan 250061, China; 3 Department of Production Engineering, Faculty of Mechanical Engineering, Koszalin University of Technology, Racławicka 15-17, 75-620 Koszalin, Poland; krzysztof.nadolny@tu.koszalin.pl; 4 School of Mechanical and Design Engineering, University of Portsmouth, Portsmouth PO1 3DJ, UK; Khaled.giasin@port.ac.uk; 5 Department of Mechanical Engineering, Main Campus-Kapurthala, IK Gujral Punjab Technical University, Punjab 144603, India; shubham543sharma@gmail.com

**Keywords:** surface belt grinding, machining, cutting speed, surface roughness, material removal rate (MRR)

## Abstract

This paper presents the research results of one of the main technological parameters of belt grinding, i.e., the cutting speed while machining corrosion- and heat-resistant, structural carbon and structural alloy steels, aluminum, and heat-resistant nickel alloys. Experimental and analytical methods are used to establish the dependence of the output parameters of surface belt grinding on the cutting speed and tool characteristics. An analytical model, considering the physical and mechanical properties of the grinding belt (strength depending on the base and bond; the thermal conductivity; the type of grinding operation) and the machined material, is created to determine the belt grinding speed. The output parameters, such as the arithmetic mean of the surface roughness (Ra) and the material removal rate (MRR) during the belt grinding of steels, heat-resistant and light alloys, have been studied. Based on the empirical dependencies of the belt grinding parameters, the model was developed for the selection and setting of the cutting speed of belt grinding for the aforementioned alloys, taking into account the type of operation, the type of the machined material, and the main characteristics of the sanding belt.

## 1. Introduction

The use of abrasive tools on a flexible base, including grinding belts, in leading industries (aerospace [1], machinery [2,3], automotive [4], etc.) is significant and accounts for about 40% of the total volume of abrasive tool use. Cloth grinding belts are most widely used, with a prevalence of up to 80% [5,6]. The most important precision parameter for belt grinding is surface roughness. Wang et al. [7] evaluated grain wear by the parameters of 3D surface roughness Sz (maximum height), Sdq (root-mean-square gradient) and Spc (arithmetic mean peak curvature) for dry belt grinding on AISI52100 hardened steel. Luo et al. [8] studied the influence of the grinding force, grinding speed, feed rate, and grain size on the surface roughness during the belt grinding of a titanium alloy. Li et al. [9] obtained the optimal values of surface roughness and material removal rate (MRR) for the belt grinding of 45 steel. Xie et al. [10] proposed a method for predicting and modeling the topography of a grinding surface based on a graphics processor (GPU). Van Gorp et al. [11] investigated the effect of operating parameters on surface roughness during belt grinding of hardened steels. Gowri et al. [12] investigated the belt grinding process of 304 stainless steel and optimized the output parameters. Zou et al. [13] investigated robotic belt grinding techniques for precision machining of aluminum blades and provided a machined surface roughness of less than 0.4 μm. Xiao et al. [14] conducted a comprehensive study of the influence of the relative direction of grinding on the process of belt grinding. They showed that up grinding resulted in higher roughness parameters and higher residual stresses compared to down grinding. Qu et al. [15] proposed a chip thickness model to predict the surface roughness of a part during the belt grinding of Ti-6Al-4V alloy. Stadnik et al. [16] showed the advantages of belt grinding and the possibility of obtaining a surface roughness (Ra) of 0.04–0.008 when machining steels and alloys. Bratan et al. [17] proposed a probabilistic model of the belt grinding process, including its main output parameters. Wang et al. [18] focused on optimizing the parameters of the belt grinding of manganese bronze to improve surface roughness, belt wear, and MRR. However, these papers are generally experimental and do not disclose the use of technically sound recommendations for their application and, prior to application, for the selection and setting of the main parameter of machining—the cutting speed of the grinding belt. It would be useful to obtain similar recommendations for surface belt grinding for a range of material groups such as steels, light alloys, and heat-resistant alloys.

Although surface belt grinding is found in the works of researchers, standards for belt cutting modes have not been developed and recommendations summarizing the dependences of the surface grinding of metals have not been presented, and recommendations for individual parameters are often derived from “advertising” literature or are not scientifically grounded. Therefore, in each specific case, the machinist is forced to experimentally determine the suitability of the belt and determine the cutting speed.

The inconsistency of recommendations is caused by the poor elaboration of the mechanism of influence of the main belt grinding parameters and the properties of the belt on output parameters. Currently, the literature poorly reflects the applied indicators, which are recommended for the selection and use of grinding belts.

**The purpose of this work** is to improve the grinding of metals using cloth grinding belts by developing technically justified recommendations to determine the cutting speed for cloth grinding belts during the surface grinding of steel, aluminum and nickel alloys. The following objectives were considered while performing this scientific work:

- To study and establish the dependences on the cutting speed of grinding belts and the most widely used characteristics in metalworking;
- To develop and select the resulting surface roughness and material removal rate, reflecting the influence of the cutting speed on grinding different alloys with cloth grinding belts;
- To develop recommendations for the selection and setting of the cutting speed during metalworking with cloth grinding belts.

## 2. Materials and Methods

### 2.1. Theoretical Provisions

Belt grinding in abrasive machining holds a specific place. The kinematics and dynamics of abrasive machining with grinding belts, and the accompanying physical phenomena, occupy an intermediate position between grinding with abrasive (i.e., hard, almost non-deformable) wheels and machining with loose abrasive [19] (see Table 1).

A distinctive feature of grinding belts is that during the cutting process, their design and properties provide for a significant elastic movement of abrasive grains from their static position in a direction perpendicular to the working surface of the tool. Due to the flexibility of grinding belts during machining, it is often impossible to preset a certain cutting depth, such as, for example, when grinding with abrasive wheels on a ceramic or bakelite bond. The necessary cutting conditions are created by setting the correct machining modes and, first of all, the main technological parameter—the cutting speed [19].

In studies of the grinding process, a significant inconsistency in the information on the selection of technological parameters of the grinding of steels and alloys, including the selection of the cutting speed, was observed. In [16,20], the recommended speed of grinding stainless steels is 17.5–25 m/s for roughing and 20–29 m/s for finishing; in [21], the recommended speed is 10–25 m/s for roughing and 0.5 m/s for finishing. In [22], identical speeds of 20–35 m/s are recommended for grinding heat-resistant steels, aluminum alloys, and hardened steels. In [9], a grinding speed of 3–9 m/s is proposed for belt grinding of steel 45.

Abrasive belts, made of new grinding materials (Cubitron [23], Cubicut [24], Alundum [25,26], etc.) with wear-resistant bonds and new polyester bases, allowing one to bring belt grinding closer to milling, are widely advertised. The main methodological provisions for the selection of the quality content of the indicators necessary to assess the performance properties of the grinding belt are given in [19,27]. The methodology includes taking into account the main factors, namely the grinding scheme, the type of machined material, the type of belt grinding stage, the parameters of the characteristics of the grinding belt, and the parameters of the grinding mode. The indicators are given for a unit of the belt’s working surface (or the contact between the belt and the workpiece), making the output parameters comparable.

When developing output parameters, the selected indicators were differentiated by the designation of the tool to correctly reflect the physical essence of belt grinding. Thus, the assessment of the operating properties of the tool for the roughing and finishing operations, and for polishing, were differentiated based on the requirements for these operations, as the main purpose of roughing is to remove the allowance in the optimum minimum time. The main purpose of finishing and polishing is to achieve the required surface quality in the optimum minimum time. On this basis, we selected indicators per the type of operation.

The physical laws of abrasive machining established by Korchak are reflected in the analytical relationship between the output parameters and the volume of the material removed when machining a workpiece ($Q_{mach}$) [28]:

(1) $$Q_{mach}=\frac{P_{y}\cdot v_{c}\cdot \tau }{\sigma_{i}\cdot K_{1}}-K_{2}\cdot f\left(\tau \right)\cdot v_{c}$$

where: *P_y_* is the cutting force directed along the normal to the machinable surface; $v_{c}$ is the cutting speed; τ is the time of machining; *σi* is the stress intensity in the shear zone of the material being machined (the stress intensity is a function of the strain intensity, ε, the strain rate, $\dot{\varepsilon }$, and the temperature, $T^{0}$, of the material—$\sigma_{i}=f\left(\varepsilon ,\dot{\varepsilon },T^{0}\right)$) [29,30]; *K*_1_ and *K*_2_ are the coefficients taking into account the geometry of the abrasive grains; *f (*τ) is the actual contact area between the tool and the workpiece, which changes during machining.

It follows from Formula (1) that the process of material removal is a function of the radial force, the cutting speed, the characteristics of the tool, and the machinable material. That is, the first requirement for the indicators is met if they reflect such basic indicators as material removal, the tool durability (and wear), and the limiting parameters: the cutting speed and the quality of machining. The second requirement for the indicators is met if the formulas take into account the dominant factors and the parameters, given the operating conditions of the tool. That is, to ensure the comparability of the indicators, it is necessary to have a unit of contact and make them speed-specific. The complex of indicators of the grit paper and the belt for roughing and finishing operations include comparable quantitative characteristics of machining, which reflect the high-speed interaction of contacting surfaces; for example, the reduced cutting ability (performance index) ($q_{Per}$) [19,27,31]. The physical meaning of the characteristic is $q_{Per}$ stock removal (materials rate removal (MRR)) from the workpiece per unit of work, which is given by the formula:

(2) $$q_{Per}=\frac{\sum_{1}^{n}q_{i}}{\tau \cdot P_{c}\cdot v_{c}}$$

where: $q_{i}$ is the material removal over the i-th grinding period; *P_c_* is the force of clamping the tool to the workpiece. To ensure the comparability of the indicators of the tool processes parameters for the pressure, the workpiece speed, the characteristics of the machinable workpiece, and the initial roughness, were investigated.

Many influencing factors affect the results of the grinding operation. In this study, several of those factors have been selected, namely: the grinding scheme (surface belt grinding with a contact roller), the type of material being processed (steels, aluminum and nickel alloys used in the same type of mechanical engineering operations and other leading industries), the type of processing (preliminary and the final allowance), parameters of the characteristics of the sanding skin of the belt (grain size, bond, base, and abrasive material), parameters of the grinding mode (clamping force of the tool and workpiece, belt speed, workpiece speed, etc.). The methodological provisions of the work were tested in laboratory conditions on an experimental stand, simulating manufacturing conditions. Approximate experimental dependences were obtained, recommendations were developed depending on the above parameters. Production tests were carried out to validate the established dependencies and recommendations. The convergence of production and laboratory tests has been determined.

### 2.2. Experimental Details

**Figure 1:**
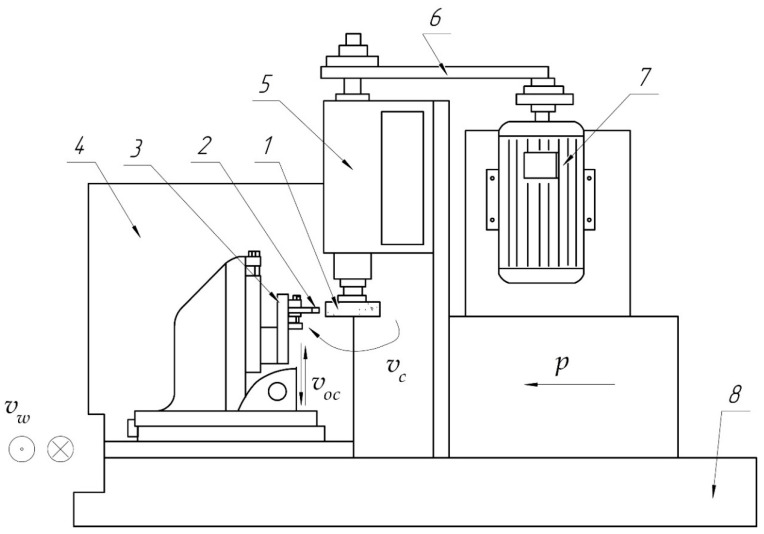
Belt grinding scheme: 1—grinding belt; 2—workpiece; 3—workpiece clamping device; 4—casing; 5—spindle unit (headstock); 6—belt drive; 7—electric motor; 8—machine stand; *P*—pressure; *v_c_*—belt speed; *v_w_*—workpiece speed; *w_os_*—vertical oscillation frequency.

The grinding belts were tested with operations according to the belt grinding scheme most common for machining—a surface belt grinder with a contact roller (see Figure 1). The experiments were carried out on a cloth sanding belt of the most widely used characteristics with a natural and synthetic bond when grinding metals of different machinability set in [27]. The experiment used a range of grinding speeds limited by the strength of the belt and reasonable durability. The restrictions allowed the application of the following full factorial experiment (FFE) method:

(3) $$N=q_{y}{}^{k}=2^{3}$$

where: *N* is the number of experiments; $q_{y}$ is the number of levels; *k* is the number of factors.

The design matrix for the selected case of FFE = 2^3^ is presented in Table 2.

The transition from the code expression of the factor to the natural value of the *i*-th factor (*X_n_*) is set by the formula [19]:

(4) $$X_{n}=X_{0}+X_{i}\cdot \delta$$

where: $X_{0}$ is the natural value of the factor at the zero level; $X_{i}$ is the code value of the *i*-th factor; δ is the natural value of the variation interval. For the experimental studies, an IS-78 model stand (Russia, Chelyabinsk, ChOZ plant (Chelyabinsk Experimental Plant)), designed based on an upgraded 3110M circular grinder (Tbilisi Grinding Machine Plant, Tbilisi, Georgia)), was used (see Figure 1). The contact length depends on the machinability group, the type of machining (primary machining or finishing), the material, and the type of contact roller (grooved, smooth). A grinding belt was a cloth-based grit paper made of 15A Brown Aluminum Oxide with F60 grain size on a synthetic bond as per GOST 27181 (Russian Standard). The experimental studies provided for grinding materials of different groups, including cast aluminum alloy AK5M2/AL3V (an analog of A319.0) as per GOST 1583, structural alloy steel 30KhGSN2/30KhGSNA as per GOST 1583, structural carbon steel 45 (an analog of AISI 1045) as per GOST 1050, corrosion-resistant and heat-resistant stainless steel Kh18N10T (an analog of AISI 321) as per GOST 5632, and heat-resistant nickel alloy KHN77TYUR (an analog of AISI 321) as per GOST 5632. Table 3 shows the chemical composition and physical and mechanical properties of these alloys [27].

The experimental study of the belt operation depending on the cutting speeds was carried out in the range of 9–35 m/s. The tests were carried out with a constant radial force, at pressures of 0.2, 0.4, and 0.8 MPa, when machining materials with a large machinability difference: steels 45, 30 KhGSN2, and Kh18N10T; alloys AK5M2 and KhN77TYuR. The following conditions were used in the experiment: the vertical oscillation frequency ($\omega_{os}\;$= 200 rpm), the longitudinal feed rate ($v_{s}\;$= 0.058 m/s), and the oscillation value ($A_{os}\;\;$= 3 mm). The roughness of the machined surface (arithmetic mean deviation of the assessed profile R_a_) was measured using a surface roughness profilometer with a unified electronic AP system, model 263 (Proton JSC, Orel, Russia).

## 3. Results and Discussion

**Figure 2:**
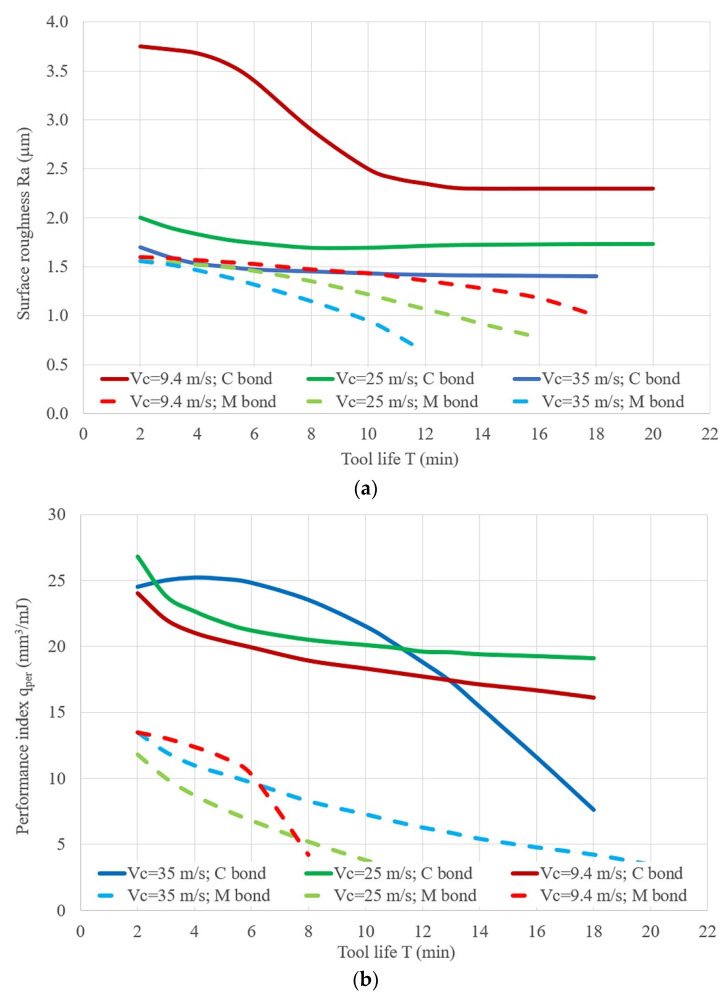
The dependence of the reduced cutting ability (**a**) and the roughness of the machinable surface (**b**) on the cutting speed during belt grinding: $v_{c}\;$= 35 m/s; $v_{c}\;$= 25 m/s; $v_{c}\;$= 9.4 m/s; for the belt on a C synthetic bond and on an M natural bond: workpiece material: steel 45; workpiece speed *v_w_* = 0.058 m/s; vertical oscillation frequency *w_os_* = 200 mm^−1^; the value of the vertical oscillation *Aos* = 3 mm; grit = F60.

Figure 2 shows the change in the cutting ability (see Figure 2a) and roughness of the machined surface (see Figure 2b) for the operation time of the 14A25 grinding belt, on a synthetic bond (C) and a natural bond (M), at different grinding speeds of steel 45 (at a pressure of 0.4 MPa). The analysis showed that the initial MRR and the durability of the belts reach their maximum values at $v_{c}\;$= 25 m/s, then at $v_{c}\;$= 35 m/s, and their minimum values at $v_{c}\;$= 9.4 m/s.

An analysis of the data allowed showed that the performance index (cutting ability) $q_{Per}$, mm^3^/min for the durability period is higher at $v_{c}\;$= 35 m/s, but the grinding belt loses its cutting properties faster than at a speed of 25 m/s. The performance index $q_{Per}$ at a machining speed $v_{c}\;$= 25 m/s is higher than when grinding at a speed $v_{c}\;$= 35 m/s. Besides, as shown by the analysis, the highest achieved surface roughness corresponds to the lowest grinding speeds. A comparison of the experimental dependences of $q_{Per}$ overtime for the grinding belts on C and M bonds showed that the dependences of $q_{Per}$ for the grinding belts with C bonds have a flatter arrangement, are longer, and reach higher values. These indicators are explained by the stronger retention of the abrasive grain by the synthetic C bond and, therefore, during grinding, the abrasive grains can work for longer, and the belt loses its performance mainly due to the dulling of the abrasive layer.

**Figure 3:**
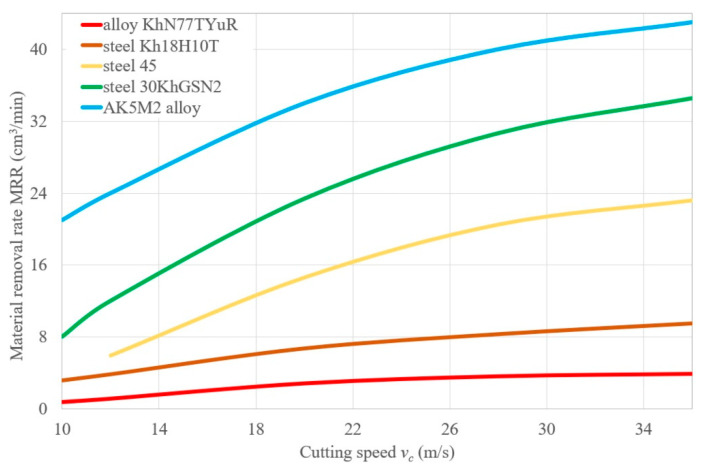
The dependences of the material removed rate (MRR) on the cutting speed of belt grinding of various materials: alloy KhN77TYuR; steel Kh18H10T; steel 45; 4—steel 30KhGSN2; 5—AK5M2 alloy: workpiece speed *v_w_* = 0.058 m/s; vertical oscillation frequency *w_os_* = 200 mm^−1^; the value of the vertical oscillation *Aos* = 3 mm; grit = F60.

Figure 3 illustrates changes in the material removal rate (MRR) depending on the grinding speed (at a pressure of 0.4 MPa) of various materials with the 14A25C belt. The results indicated that an increase in the cutting speed leads to an increase in the number of the grain contacts with the machinable surface per unit of time, and to an increase in the MMR. When grinding different metals, the maximum MRR is observed at the highest tested speed of 35 m/s (at pressures of 0.4 and 0.2 MPa) [27]. This is explained as follows: with an increase in the grinding speed, the cutting ability of the belt increases because more working grains take part in the material removal per unit of time [27,32,33]. A possible factor which is affecting the decrease in MRR of the belt at a grinding speed of 35.3 m/s could be the increased wear of the belt [34]. With an increase in the speed, there is a sharp increase in the specific loads on single grains, which wear out more quickly, and the wear on the binder also increases [3,35]. The M bond, being relatively softer (with lower hardness values (*N_max_*)), does not offer sufficient resistance to wear with increasing speed [36].

**Figure 4:**
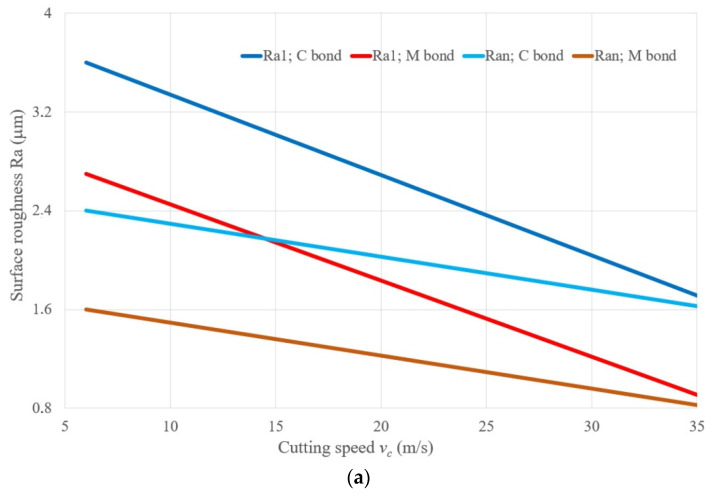

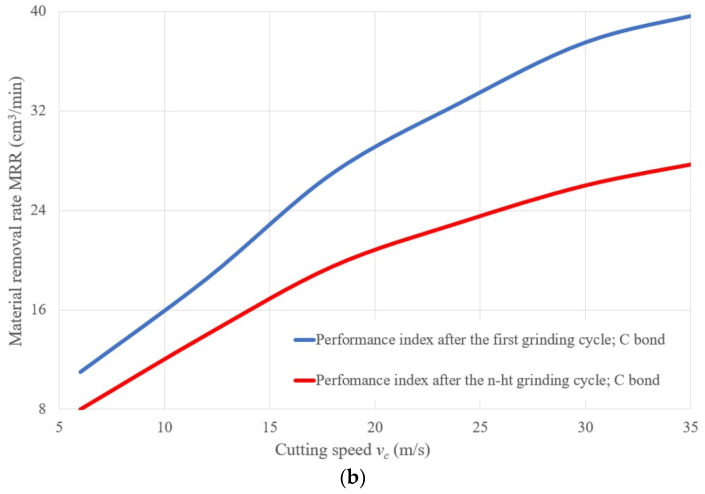
The dependencies of the surface roughness Ra (**a**) and the material removal rate (MRR) (**b**) on the cutting speed; for the belt on a C synthetic bond: workpiece material steel 45; workpiece speed *v_w_* = 0.058 m/s; vertical oscillation frequency *w_os_* = 200 mm^−1^; the value of the vertical oscillation *Aos* = 3 mm; grit = F60.

Table 4 gives the values of the output parameters determined during the tests—material removal rate (MRR, cm^3^/min), performance index ($q_{Per}$, mm^3^/mJ), and tool wear ($V_{B}$, g)—during the belt grinding of various materials at different cutting speeds ($v_{c}$). The data confirm the above dependences of the output parameters during belt grinding of various materials on the cutting speed. The changes in the surface roughness (Ra), when machining different materials at different cutting speeds, after the first grinding cycle ($Ra_{1}$) and after the *n*-th grinding cycle ($Ra_{n}$), are shown in Figure 4 and correspond to the changes in *q*: the worst roughness is obtained at the lowest speed *(*$v_{c}\;$= 9.4 m/s), the best at the highest speed ($v_{c}\;$= 35.3 m/s). The experimental dependences were obtained during grinding with a 14A25M belt and at a pressure of 0.2 MPa. The analysis showed that the influence of the belt speed on the roughness ($Ra_{1}$ and $Ra_{n}$) is significant and expressed by a correlation dependence with a high correlation coefficient from νR = −0.94 ± 0.02 to νR = 0.97 ± 0.02, and is described by:

(5) $$Ra_{1}=a_{1}-b_{1}\cdot v_{c}$$

(6) $$Ra_{n}=a_{n}-b_{n}\cdot v_{c}$$

Table 5 gives the values of the coefficients $a_{1}$, $a_{n}$, $b_{1}$ and $b_{n}$ in Formulas (5) and (6) for representatives of the material groups, depending on their machinability with a grinding belt (defined in [19]), and the correlation coefficients.

The dependences of the output parameters on the change in the grinding speed remain for the entire range of the tested pressures p = 0.2–0.8 MPa, only their nature changes: from smooth at small p and $v_{c}$, to steeper ones at higher p and $v_{c}$.

For example, for the 14A25C belt:

at p = 0.2 MPa

$Ra_{1}=4.40-0.06\cdot v_{c}$ and $Ra_{n}=2.98-0.06\cdot v_{c};$

at p = 0.4 MPa

$Ra_{1}=4.29-0.07\cdot v_{c}$ and $Ra_{n}=2.64-0.04\cdot v_{c}.$

The experiments show that the tool durability decreases by 10–60%, depending on the characteristics of the belt grit paper and the machinable material, with a grinding speed 1.4 times above 25 m/s. When testing at speeds of over 35.3 m/s, depending on the material and the pressure, thermal damage and burnout of the natural glue occur due to the increased wear and a sharp increase in the temperatures in the contact zone. To differentiate groups of grit paper strength by the breaking strength, which changes with the grain size, in this study, the base of the grit paper was, first of all, technologically selected according to its grain size [37]. The processing of a significant amount of the experimental data obtained during the tests allowed us to derive an empirical formula:

(7) $$v_{c}=a_{b}-b_{b}\cdot N$$

where: $a_{b}$ and $b_{b}$ are the coefficients of the group of material machinability with a belt for roughing and finishing; N is the grain size.

The coefficients $a_{b}$ and $b_{b}$ change depending on the material machinability group and the type of machining (roughing, finishing); the latter is determined by the size of the allowance (*A*), and depends on the change in *N* and the strength group of the grit paper. Table 6 gives examples of Formula (6) for each group of machinable materials and the machining type with the correlation coefficients of the function ρ = f (*N*). The correlation coefficients show the influence of *N* on $v_{c}$ and are in the range ρ = 0.92 ± 0.006. To develop recommendations for the belt grinding speed, we proceeded primarily from the physical and mechanical properties of the grinding belt (the strength depending on the base and bond) the thermal conductivity, the type of grinding operation, and the machinable material. An analysis of the experimental data made it possible to derive an empirical design model to determine the belt grinding speed ($v_{c}$) depending on these factors, represented by the following exponential function [37]:

(8) $$v_{c}=0.1\cdot \sigma_{p}{}^{k_{v}}$$

where, $\sigma_{p}\;$is the tensile strength of the grinding belt, N/mm^2^, $k_{v}$ is an exponent taking into account the machinable material, the type of operation, the properties of the grit paper (bond type and thermal conductivity), depending on the base, the grain size, and the belt grinding speed.

The tensile strength of the grinding belt in the longitudinal direction ($\sigma_{p}$) (Formula (7)) formed eight groups for standard cloth bases, taking into account the data of national standards (GOST 5009, GOST 13344, GOST 27181), and is shown in Table 7.

**Figure 5:**
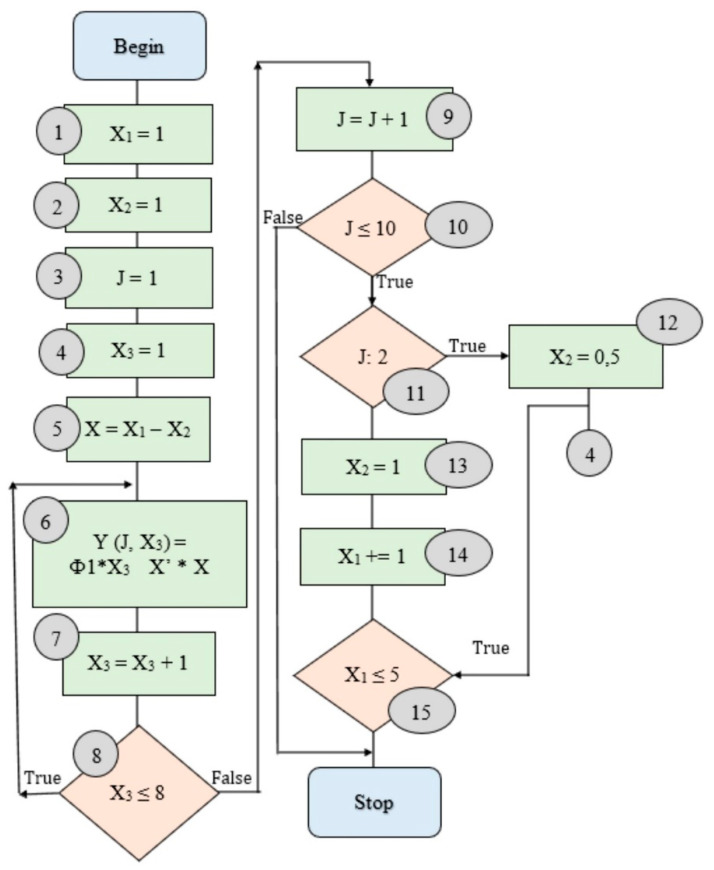
A block scheme of selecting the belt grinding speed: *Y* is the speed of the grinding belt *v_c_*, m/s; *Y* = f (*X*_1_,*X*_2_,*X*_3_); *X*_1*i*_ are material machinability groups ($k_{m}$); *X*_2_ is the type of machining (allowance up to 1 mm or 0.05 mm); *X*_3i_ is the belt strength groups (σ_p_).

The error of approximating the set dependences is 3.3–5.7%, which is acceptable and simplifies the calculations. The permissible belt grinding speeds, $v_{c}$, were taken as the basis to develop the recommendations. Figure 5 shows the sequence setting the speed of the grinding belt in the developed recommendations.

The block diagram in Figure 5 illustrates the process of choosing the speed of the sanding belt (*v_c_*, m/s) as a function of *Y* = f (*X*_1_,*X*_2_,*X*_3_), where: *X*_1_—groups of the machined material (according to the machinability factor (Co), set in work [27]); *X*_2_—the type of machining determined by the size of the allowance P up to 1.00 mm and 0.05 mm; *X*_3_—strength groups of the grinding belt (see Table 6). The group of the processed material (*X*_1_) and the type of processing (*X*_2_), as well as the belt speed (*v_c_*, m/s), is determined for a specific belt characteristic, namely, with the set *X*_3_—the belt strength group (eight groups). At stage 5 of the block diagram, we obtain the belt speed *Y* = f (*X*_1_,*X*_2_,*X*_3_) for the next type of processing *X* = *X*_1_ − *X*_2_. In parts six to eight of the block diagram, we determine the belt speed (*v_c_*, m/s) for this group (*X*_1_) and separately for each type of processing (*X*_2_), depending on the specific characteristics of the belt, namely, with the set *X*_3_—belt strength group (≤8 groups). At stages 9–14, the transition to the determination of *v_c_*, m/s of the next group of processed material (*X*_1_ = *X*_1_ + 1) and for a certain type of processing (*X*_2_) is carried out. At stage 15, we determine *v_c_*, in m/s, for the next type of processing (*X*_2_ = 0.5), and also for each of the groups of the processed material (*X*_1_ ≤ 5). So, for example, for *X*_1_ (the third group of materials) and *X*_2_ (preliminary grinding at *X*_3_—the first group of strength *v_c_* = 14.9 m/s), if *X* = *X*_1_ − *X*_2_, we get *v_c_* = 19.8 m/s, and for the second group of strength *X*_3_ = *X*_3_ + 1, we get *v_c_* = 15.0 m/s and *X* = *X*_1_ − *X*_2_ is determined by *v_c_* = 20.0 m/s.

## 4. Summary

The research carried out allowed us to obtain the following results. The developed set of performance indicators, including those previously used in practice (material removed rate (MRR), tool life, roughness and wear) and those proposed in this work (material removed rate (MRR), reduced cutting ability (performance index), roughness for the first cycle and roughness for the n-th cycle) makes it possible to objectively evaluate the processing results of the belt grinding process. Significant statistical data of tests made it possible to derive an empirical model for calculating the speed of belt grinding from the grain size of the belt, as well as the group of metal machinability for roughing and finishing. Processing of statistical data on the dependence of the belt grinding speed on the physical and mechanical properties of the grinding belt, such as strength, depending on the base and bond, thermal conductivity, the type of grinding operation and the type of material being processed, made it possible to derive an empirical design model for determining the cutting speed of a tape from these factors in the form of an exponential function. Through the equations used for calculating the dependence of the cutting speed on the grain size of the tape, the type of operation, properties of the skin, and the type of bond and thermal conductivity, were obtained for five groups of machinability. The results of experimental studies revealed the presence of dependences of the main parameter of the grinding process—the cutting speed of the belts on the characteristics of the grinding paper, the type of material being processed, and the type of operation. The established dependencies and their approximation determined the mathematical models for calculating the function of the initial and final roughness of the machined surface on the grinding speed for each group of machinability of steels and alloys and the type of belt grinding.

The dependencies of the output parameters of the belt on the main parameter of belt grinding modes—the cutting speed—are complex but mainly have extreme ranges of values. The output parameters (operating time, cutting ability, etc.) are linked by a direct functional dependence with the grinding speeds (i.e., performance increases as speeds grow) to certain values, after which the increase stops. The roughness of the machined surface is inversely related to the grinding speed; the roughness decreases with an increase in the grinding speed.

The experiments established the maximum values of the output parameters at the optimal values of the grinding speed in combination with certain set test conditions.

The influence of the belt speed on the output parameters during grinding, depending on a combination of factors and developed recommendations for setting the grinding belt speed, was established. For rough grinding, the recommended belt speed is generally 20–30% lower than for finishing. With a decrease in the allowance, the recommended machining speed increases, which allows us to increase the productivity of the grinding and reduce the roughness of the machined surface, but belt wear also increases. The shape of the machinable workpiece also affects the change in the speed. It is recommended to grind flat workpieces at slightly lower speeds than cylindrical ones.

As a result of studying surface belt grinding, we developed recommendations for the belt speed based on the requirements for the type of operation and depending on the type of the material, to achieve the best output parameters and optimal combinations of material removal rate (MMR), belt wear, and the roughness of the machined surface.

## 5. Conclusions

The relevance of the work is justified by the aggravation of competition in market conditions, which requires manufacturers of machine-building products to look for reserves in order to increase production efficiency. The measures developed in this study are aimed at increasing the efficiency of the enterprise, namely, the operation of belt grinding, paying particular attention to the issue of choosing reasonable and rational technological conditions for their implementation. The successful solution of this problem allows the creation of a reserve for further increasing the technical and economic efficiency of manufacturing without additional labor-intensive and material costs.

As a result, the optimal main technological parameter of belt grinding—belt speed— was identified based on experimentally established dependences on real process factors.

An analytical model for determining the value of the belt grinding speed was created, taking into account the physical and mechanical properties of the grinding belt (the strength depending on the base and bond), thermal conductivity, the type of the grinding operation, and the machinable material.

Groups of values were formed for the tensile strength of the grinding belt in the longitudinal direction for standard cloth-based grit paper, taking into account national (Russian) standards, and they were included in the recommendations for the belt grinding speed.

The provisions for the calculation, selection, and application of the output parameters were determined to assess belt grinding. Based on the results, we collected the statistics of the output parameters according to the dependences on the cutting speed of the characteristics of the grinding belt, the type of machining, and the machinable material. The experimental studies determined the empirical dependences of the belt grinding speed for groups of steels and alloys with a grinding belt on the characteristics of the grinding belt, the type of machining. The error in approximating the dependences was 3.3–5.7%. We determined the significant influence of the belt speed on the roughness of the machined surface and described it by linear functions and the correlation dependence with a high correlation coefficient (from 0.94 ± 0.02 to 0.97 ± 0.02).

Based on these empirical dependences, we developed recommendations for the belt speed in ranges covering the most widely used grinding schemes (primarily, surface grinding), for the machinability groups of steels and alloys, the main characteristics of the tool, and the type of operation provided by the technical parameters of the equipment.

The recommendations for the belt speed for grinding and finishing allow us to guarantee the optimal performance of grinding operations while ensuring a specified surface quality under variable machining conditions.

The recommendations are of significant practical value and can be used at leading abrasive enterprises and enterprise consumers.

## Figures and Tables

**Table 1:** A comparison of belt grinding with wheel and loose abrasive grinding.

Type of Grinding	Operating Speed, m/s	Contact Temperature, °C	Accuracy Degree	Surface Roughness Ra, µm	Type of Residual Stresses after Machining
Abrasive wheel	10–100	700–1200	5	0.08	Stretch
Belt	10–30	400–800	5,6	0.02	Compression
Loose abrasive	2–5	200–300	5	0.01	Compression

**Table 2:** The design matrix of the full factorial experiment.

Experiment Number	X1	X2	X3
1	−1	−1	−1
2	+1	−1	−1
3	−1	+1	−1
4	+1	+1	−1
5	−1	−1	+1
6	+1	−1	+1
7	−1	+1	+1
8	+1	+1	+1

**Table 3:** The chemical compositions and physical and mechanical properties of materials. Adapted with permission from ref. [27].

Material Group	Workpiece Material	Chemical Composition, %																		Physical and Mechanical Properties			
		Carbon, C	Silicon, Si	Manganese, Mn	Nickel, Ni	Sulfur, S	Phosphorus, P	Chromium, Cr	Cerium, Ce	Titanium, Ti	Boron, B	Lead, Pb	Iron, Fe	Aluminum, Al	Copper, Cu	Arsenic, As	Zinc, Zn	Magnesium, Mg	Other Impurities	Yield Stress, *σ_y_,* MPa	Ultimate Stress, *σ_D_*, MPa	Density, *ρ*, kg/m^3^	Hardness, HB
Aluminum alloy	AK5M2/AL3V	—	4–6	0.2–0.8	to 0.5	—	—	—	—	0.05–0.2	—	—	to 1.3	85.9–94.05	1.5–3.5	—	to 1.5	0.2–0.8	total 2.8	162	—	2900	70
Structural alloy steel	30KHGSN2 (30KHGSNA)	0.27–0.34	0.9–1.2	1–1.3	1.4–1.8	to 0.025	to 0.025	0.9–1.2	—	—	—	—	≈95	—	to 0.3	—	—	—	—	1375	1620	7770	255
Structural carbon steel	45	0.42–0.5	0.17–0.37	0.5–0.8	to 0.25	to 0.04	to 0.035	to 0.25	—	—	—	—	≈97	—	to 0.25	to 0.08	—	—	—	355	600	7826	207
Corrosion- and heat-resistant stainless steel	KH18N10T	to 0.12	to 0.8	to 2.0	9–11	to 0.02	to 0.035	17–19	—	0.6–0.8	—	—	≈68	—	—	—	—	—	—	196	510	7920	179
Heat-resistant nickel alloy	KHN77TYUR	to 0.07	to 0.6	to 0.4	70.076–77.4	to 0.007	to 0.015	19–22	to 0.02	2.4–2.8	to 0.01	to 0.001	to 1	0.6–1	—	—	—	—	—	650	1000	8200	255–321

**Table 4:** Output parameters of belt grinding of various materials obtained at various cutting speeds ($v_{c}$).

Material Grade	$P_{Y},\;N$	$v_{c}\;=\;9.4\;m/s$			$v_{c}\;=\;25.0\;m/s$			$v_{c}\;=\;35.3\;m/s$		
		MRR, cm3/min	$q_{Per},$ mm^3^/mJ	$V_{B},\;g$	MRR, cm3/min	$q_{Per},$ mm^3^/mJ	$V_{B},\;g$	MRR, cm3/min	$q_{Per},$ mm^3^/mJ	$V_{B},\;g$
AK5M2	56.9	48.28	2.41	1.67	144.40	7.92	0.95	170.40	8.52	1.20
	34.3	28.88	1.44	1.45	63.10	3.15	0.87	86.40	4.32	1.00
30KhGSN2	56.9	10.28	0.51	1.55	52.08	2.60	0.88	67.02	3.35	1.20
	34.3	8.65	0.43	0.80	27.41	1.37	0.73	30.59	1.53	1.55
45	56.9	11.47	0.57	1.00	34.40	1.72	0.70	42.60	2.24	7.60
	34.3	6.86	0.34	–	14.99	1.13	0.80	21.60	1.18	0.75
KhI8NI0T	56.9	2.55	0.13	1.25	6.82	0.67	1.90	13.30	0.85	2.70
	34.3	2.38	0.12	–	6.62	0.33	1.30	8.16	0.53	1.87
KhN77TYUR	56.9	1.65	0.08	1.25	7.65	0.39	1.65	5.53	0.28	2.00
	34.3	1.30	0.065	–	5.06	0.25	1.15	4.24	0.21	1.40

**Table 5:** Coefficients of the empirical function of the initial and final surface roughness depending on the cutting speed.

Grade of Metal	$Ra_{1}=a_{1}-b_{1}\cdot v_{c}$ $Ra_{n}=a_{n}-b_{n}\cdot v_{c}$				Correlation Coefficient
	$a_{1}$	$a_{n}$	$b_{1}$	$b_{n}$	
AK5M2	3.4029	-	−0.0149	-	−0.9545
	-	1.6489	-	0.0069	−0.9703
30KhGSN2	2.9987	-	–0.0038	-	0.9891
	-	2.4979	-	−0.0398	0.9878
45	2.2688	-	0.0223	-	−0.9911
	-	1.0996	-	−0.0088	−0.9840
Kh18N10T	1.7021	-	−0.0171	-	0.9561
	-	0.8252	-	−0.0071	0.9632
XH77TЮP	1.4093	-	−0.0219	-	−0.9410
	-	0.6428	-	−0.0085	−0.9980

**Table 6:** Empirical linear functions of the dependence of the grinding speed on the grain size of the belt.

Machinability Groups	$\mathrm{Finishing}\;\mathrm{Allowance}\;\Delta_{i}\;\leq \;0.05\;\mathrm{mm}$		$\mathrm{Roughing}\;\mathrm{Allowance}\;\Delta_{i}\;\geq \;0.05\;\mathrm{mm}$	
	Dependency Equation	Correlation Coefficient	Dependency Equation	Correlation Coefficient
1	$v_{c}$ = 31.825 + 9.545 N	0.9260	$v_{c}$ = 26.581 + 9.988 N	0.9156
2	$v_{c}$ = 27.819 + 9.641 N	0.9243	$v_{c}$ = 23.953 + 9.420 N	0.9238
3	$v_{c}$ = 19.511 + 12.216 N	0.9235	$v_{c}$ = 14.550 + 12.211 N	0.9233
4	$v_{c}$ = 16.585 + 12.179 N	0.9214	$v_{c}$ = 11.521 + 12.145 N	0.9282
5	$v_{c}$ = 12.7 + 12.115 N	0.9201	$v_{c}$ = 9.3 + 12.105 N	0.9187

**Table 7:** Grinding belt strength groups used in the recommendations for setting the cutting speed.

Cloth Base of the Belts Grit Paper as Per GOST 5009, GOST 13344, GOST 27181	Grinding Belt Strength Groups	
	Group Number	Limits of the Tensile Strength in the Longitudinal Direction, N
Light gray twill #1 Whole-colored semi-two-thread cloth Extra-light gray twill Extra-light whole-colored twill	1	935–1039
Medium whole-colored twill #1 Light gray twill #2	2	1040–1144
Medium gray twill #1	3	1145–1249
Medium whole-colored twill #2 Medium whole-colored twill #1	4	1250–1354
Weighted whole-colored twill #1 Medium gray twill #2 Medium whole-colored twill #2	5	1355–1459
Weighted whole-colored twill #2 Weighted gray twill #1	6	1460–1564
Weighted whole-colored twill #1 Weighted gray twill #2	7	1565–1669
Medium gray scoured twill #2 Weighted gray twill #2 Weighted whole-colored twill #2 Special durable twill Weighted gray scoured twill #2 Weighted gray desized twill #2	8	1670–1774

## Data Availability

All data generated or analysed during this study are included in this article.

## Nomenclature

*f* (τ)	Actual contact area between the tool and the workpiece
$\Delta_{i}$	Allowance
*A_os_*	Amplitude of the vertical oscillation
*Ra*	Arithmetic mean deviation of the assessed profile (surface roughness)
*_Vb_*	Belt speed
*P_c_*	Clamping force
$X_{i}$	Code value of the i-th factor
*v_c_*	Cutting speed
*ρ*	Density
ß	Deviation angle of the cutting grain from the vertical position during grinding
$k_{v}$	Exponent taking into account the machinable material
$V_{B}$	Grain blunting area (flank wear)
GPU	Graphics processor
HB	Hardness
$v_{s}$	Longitudinal feed rate
*Q*	Material removal
MRR	Material removal rate
$q_{i}$	Material removal rate over the i-th grinding period
$X_{0}$	Natural value of the factor at the zero level
δ	Natural value of the variation interval
*N*	Number of experiments
*k*	Number of factors
$q_{y}$	Number of levels
τ	Operating time of the tool till the resistance criterion (tool life)
$q_{Per}$	Performance index
*p*	Pressure
*P_y_*	Radial cutting force
ε	Strain intensity
$\dot{\varepsilon }$	Strain rate
*σ_i_*	Stress intensity in the shear zone of the material being machined
$Ra_{1}$	Surface roughness after the first grinding cycle
$Ra_{n}$	Surface roughness after the n-th grinding cycle
$K_{1}$ $;\;K_{2}$	The coefficients accounting for the geometry of the cutting part of abrasive grains and the nature of metal yielding in the deformation zone
$T^{0}$	Temperature
$\sigma_{p}$	Tensile strength of the grinding belt
$V_{B}$	Tool wear
*X_n_*	Transition from the code expression of the factor to the natural value of the i-th factor
*σ_D_*	Ultimate Stress
*w_os_*	Vertical oscillation frequency
*_Vw_*	Workpiece speed
*σ_y_*	Yield Stress
*X*_1_	Groups of the machined material
*X*_2_	The type of machining determined by the size of the allowance P up to 1.00 mm and 0.05 mm
*X*_3_	Strength groups of the grinding belt
